# Automated deep learning–radiomics pipeline for non-calcified coronary plaque detection using non-contrast calcium score CT

**DOI:** 10.3389/fcvm.2026.1794024

**Published:** 2026-06-01

**Authors:** Wen Chen, Qing Tao, Can Chen, Su Hu, Feirong Yao, Jie Chen, Jinggang Zhang, Kejun Gu, Li Su, Wei Xing, Chunhong Hu

**Affiliations:** 1 Department of Radiology, The First Affiliated Hospital of Soochow University, Suzhou, Jiangsu, China; 2 Institute of Medical Imaging, Soochow University, Suzhou, Jiangsu, China; 3 Department of Radiology, The Third Affiliated Hospital of Soochow University, Changzhou, Jiangsu, China; 4 Edocyun Health Ltd., Suzhou, Jiangsu, China

**Keywords:** calcium score CT, coronary artery disease, coronary segmentation, deep learning, non-calcified plaque, radiomics

## Abstract

**Background:**

Coronary artery calcium score (CACS) quantifies calcification to assess coronary artery disease (CAD), but it provides insufficient warning for low-attenuation non-calcified plaques. This study proposes and validates an automated pipeline that combines deep learning and radiomics for efficient detection of non-calcified plaques in the left anterior descending artery (LAD) and right coronary artery (RCA) using non-contrast CACS.

**Methods:**

Patients undergoing coronary CT angiography for suspected CAD from two medical sites were retrospectively enrolled and categorized into lesion and control groups. LAD and RCA vessels on CACS images from the development set were manually annotated to train deep learning-based segmentation models for automated coronary segmentation and subsequent pericoronary adipose tissue (PCAT) extraction. Radiomics models were built for LAD and RCA using three regions of interest—coronary artery, PCAT, and their combination—based on the training set. Model performance was evaluated across all datasets using receiver operating characteristic analyses, and DeLong tests were applied for pairwise comparisons.

**Results:**

The SegResNet models achieved optimal performance in coronary segmentation. Radiomics models for predicting non-calcified plaques demonstrated moderate to good vessel-level diagnostic performance, with areas under the curve (AUCs) ranging from 0.700 to 0.855 across datasets, encompassing separate LAD and RCA models and all ROI strategies. The coronary artery and combined-region models generally outperformed or matched the PCAT model, with comparable AUCs between them in most settings.

**Conclusions:**

The automated pipeline enables efficient detection of non-calcified coronary plaques in CACS, with combined-region models showing promise for future use. The approach may facilitate further research and support the clinical translation of chest CT for large-scale CAD screening.

## Introduction

Coronary artery disease (CAD) is one of the most prevalent cardiovascular conditions worldwide and remains the leading cause of death in both developed and developing nations (1). This debilitating disease often leads to myocardial ischemia and sudden cardiac death, posing a severe threat to human life, health, and well-being (2). The pathophysiologic basis of CAD is atherosclerosis (3), and its gradual progression usually goes unnoticed until it manifests as acute coronary syndrome (4). Amid this course, sudden cardiac death can strike individuals who appear “healthy”, occurring unexpectedly and causing death outside of hospitals with limited opportunities for intervention (5). The silent nature of CAD progression and its potentially fatal outcomes highlight the urgent need for early screening and diagnosis to identify at-risk individuals before catastrophic events occur. However, an intelligent and automated tool for resolving this issue is still lacking, and the technical aspects associated with such methods demand urgent consideration.

With the ability to visualize plaque characteristics and coronary luminal stenosis, coronary CT angiography (CCTA) has become the preferred non-invasive diagnostic tool for CAD in clinical practice (6, 7). However, CCTA has not been widely implemented as a screening tool due to several limitations, including possible allergic reactions from iodine contrast agents, risks for patients with renal insufficiency, elevated radiation exposure, long examination times, high costs, and the need for specialized equipment (8–10). Instead, non-contrast CT, known for its safety, broader clinical applicability, and efficiency, presents promising potential for large-scale CAD screening (11). The current consensus is that the non-contrast CT coronary artery calcium score (CACS) can evaluate the presence and severity of CAD through quantifying coronary artery calcification (12). Nevertheless, CACS imaging provides insufficient warning for low-attenuation non-calcified plaques, which may be more meaningful due to their susceptibility to rupture and thrombosis, as well as their association with increased risks of adverse cardiovascular events (7).

In this context, several studies have indicated that non-calcified plaques may be inferred from coronary features on non-contrast CACS images. Thilo et al. (13) reported visual signs indicative of such plaques, whilst Kruk et al. (14) suggested that imaging patterns undetectable to the naked eye could be identified using radiomics and machine learning. These findings support the use of coronary features on CACS for non-calcified plaque detection. Additionally, pericoronary adipose tissue (PCAT) has emerged as a key biomarker of coronary inflammation—an essential driver in the development of CAD and related ischemic events (15, 16). The PCAT interacts bidirectionally with the vessel wall, allowing inflamed vessels to exert paracrine effects that induce compositional changes in PCAT (17–20). The radiomics is a technique that facilitates high-throughput extraction of quantitative features (21), which can comprehensively capture subtle structural changes in PCAT and has increasingly demonstrated its effectiveness in predicting CAD (22, 23). Furthermore, while iodine contrast agents have been shown to increase PCAT attenuation under inflammatory conditions, potentially influencing risk prediction, non-contrast CACS provides a more accurate reflection of the true attenuation of PCAT (24). Therefore, integrating radiomics with CACS presents a plausible non-invasive approach for detecting non-calcified plaques.

Lately, several studies have explored the use of CACS and radiomics for screening non-calcified coronary plaques, yielding promising results (11, 14, 25, 26). However, these studies still face several limitations. One major issue is that most are based on small, single-center samples, with their generalizability remaining uncertain. Another significant challenge is that vessel segmentation in these studies has been performed manually, a process that is both time-consuming and labor-intensive. This is particularly difficult due to the low contrast between vessels and surrounding tissues in non-contrast CT images, making it impractical for widespread clinical use. These obstacles hinder the broader dissemination of related research and its translation into clinical practice. In recent years, the rapid advancement of deep learning technologies has facilitated the automated and highly accurate segmentation of medical images, including coronary arteries (27). This makes it possible to achieve efficient segmentation of coronary arteries based on CACS. Besides, both coronary arteries and PCAT provide information related to non-calcified plaques. As highlighted by Savo et al. (28), coronary artery metrics and PCAT-derived metrics are complementary rather than redundant in CAD assessment. In this context, it is worth exploring whether using coronary arteries and PCAT as a unified approach could address the inherent difficulty of discriminating coronary arteries in non-contrast CT.

**Figure 1:**
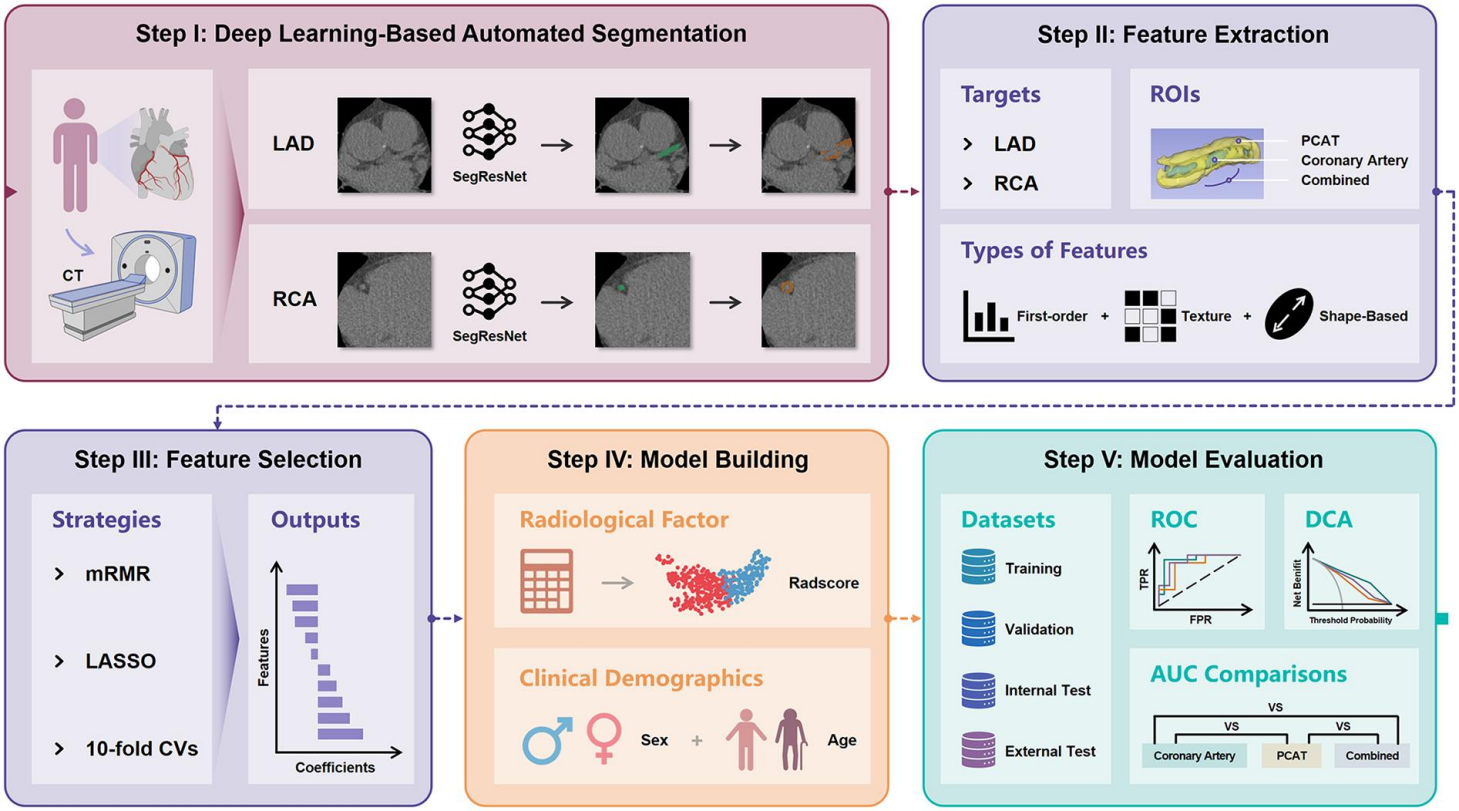
Overview of the study workflow. LAD, left anterior descending artery; RCA, right coronary artery; ROIs, regions of interest; PCAT, pericoronary adipose tissue; mRMR, maximum relevance minimum redundancy; LASSO, least absolute shrinkage and selection operator; CVs, cross-validations; ROC, receiver operating characteristic; TPR, true positive rate; FPR, false positive rate; DCA, decision curve analysis; AUC, area under the curve. Created with biorender.com.

To this end, the present study aims to propose an automated pipeline for screening non-calcified coronary plaques using CACS, where deep learning is employed for coronary artery segmentation and the diagnostic capabilities of radiomics models based on coronary artery, PCAT, and their combined region are tested on datasets from two centers with relatively large sample sizes (Figure 1). In this work, the analysis is restricted to the left anterior descending artery (LAD) and right coronary artery (RCA), as the left circumflex artery (LCX) presents challenges due to its variable anatomy, small calibre, tortuous path, and limited surrounding adipose tissue (11, 29), which may complicate reliable segmentation and plaque detection on non-contrast images. We hypothesize that our pipeline will enable efficient detection of non-calcified coronary plaques in LAD and RCA. If successful, this novel approach could guide suspected CAD patients to further CCTA examination, enhancing clinical resource utilization without additional burden, and potentially driving a paradigm transition in early screening and intervention for CAD.

## Materials and methods

### Study population

The patient enrollment flowchart is shown in Supplementary Figure 1. This study retrospectively enrolled patients who had undergone CCTA for suspected CAD from two different medical sites (site 1: The First Affiliated Hospital of Soochow University; site 2: The Third Affiliated Hospital of Soochow University). For the construction and validation of both the deep learning segmentation model and the radiomics model, we totally employed three datasets: the development set (site 1, between August 2019 and August 2021), the internal test set (site 1, between April 2021 and August 2024), and the external test set (site 2, between September 2019 and August 2020). The patients were selected based on the inclusion and exclusion criteria, and lesion and control groups were defined for each dataset. In detail, the lesion group comprised patients with non-calcified plaques in the left anterior descending artery (LAD) and/or right coronary artery (RCA) as identified by CCTA, while the control group consisted of patients with no significant abnormalities on CCTA. The inclusion criteria for the lesion group were: (1) Good image quality with adequate coronary intraluminal contrast filling; and (2) Presence of non-calcified plaques in the left LAD and/or RCA, located 10–50 mm from the coronary artery ostium. The inclusion criteria for the control group were: (1) Good image quality with adequate coronary intraluminal contrast filling; and (2) No abnormalities observed on CCTA. The exclusion criteria for both the lesion and control groups were as follows: (1) Presence of artifacts on the CACS sequence; (2) Lack of a CACS sequence with a slice thickness of 0.625 mm (pre-specified in this study for reliable assessment); (3) Calcified plaques identified in the target coronary artery; (4) Coronary artery malformation; (5) Myocardial bridge within 50 mm of the LAD; (6) Pericardial effusion; and (7) Bypass surgery, stent implantation, cardiac pacemaker, or artificial metal valve. If non-calcified plaques were present in one coronary artery and the other side was normal, the side with the plaques was included as the lesion artery, while the contralateral side was excluded. If non-calcified plaques were present in both arteries and no exclusion criteria were met, both sides were included as lesion arteries. Eligible lesions were restricted to purely non-calcified plaques on CCTA, defined as plaques with no visually detectable calcified components, and mixed plaques were excluded. The reference standard (lesion vs. control) was established using CCTA images based on: (1) initial imaging reports approved by senior radiologists, and (2) confirmation by a senior cardiovascular imaging specialist (Reader 1). Given the large vessel count and feasibility considerations, a completely random, stratified subset of 30 vessels (15 LAD, 15 RCA) was selected from the full cohort to assess reliability. A second cardiovascular imaging specialist (Reader 2), blinded to Reader 1, independently classified each vessel using the same CCTA images, achieving perfect agreement [Cohen's *κ* = 1.00; 95% confidence interval (CI): 0.77–1.00, Wilson score method]. The lower bound of the 95% CI (0.77) indicates at least substantial consistency and supports the reliability of the reference standard.

The study was conducted in accordance with the ethical standards of the institutional review board of each participating hospital, i.e., the Ethics Committee of the First Affiliated Hospital of Soochow University and the Ethics Committee of the First People's Hospital of Changzhou, which approved this study and granted a waiver of informed consent.

### CT acquisition protocol and reconstruction

For both site 1 and site 2, all image acquisitions were performed using identical types of equipment and protocols, ensuring consistency across different datasets. Both sites utilized the 256-row GE Revolution CT scanner (GE Healthcare, Milwaukee, WI, USA). Each patient first underwent a plain CT scan [electrocardiograph (ECG)-triggered CACS scan], and then proceeded to a CCTA scan. The scanning range covered from tracheal carina to 2 cm below the heart apex. The scanning parameters were as follows: tube voltage, 100 kV; tube current, 350–600 mA; and reconstruction slice thickness, 0.625 mm. The contrast agent iodixanol was administered through the cubital vein with a total volume of 50–60 mL at a rate of 4.5–5 mL/s, followed by 40–50 mL of normal saline.

To achieve optimal images, the non-contrast CT and CCTA scans were reconstructed retrospectively using iterative reconstruction techniques, and ECG editing was applied when necessary. The percentage method was used to reconstruct CCTA images, allowing for multiphasic reconstruction at five-percent intervals. The phase yielding the best image quality was selected as the final CCTA image for each patient.

### Coronary artery and PCAT segmentation

Based on the development set, we manually segmented the included LAD and RCA arteries in non-contrast CACS images using a dedicated software (Perivascular Fat Analysis Tool; Shukun Technology, Beijing, PR China). Specifically, the proximal 40 mm segments of the LAD and/or the proximal 10–50 mm segments of the RCA were traced on the CACS images (11, 18, 30), with reference to the corresponding CCTA images from the same patient. The length of each coronary artery segmentation, i.e., approximately 40 mm, was controlled using the Major Axis Length real-time display function in the software. The regions of interest (ROIs) of the vessels were carefully delineated by one experienced radiologist and supervised by another to ensure precision.

**Figure 2:**
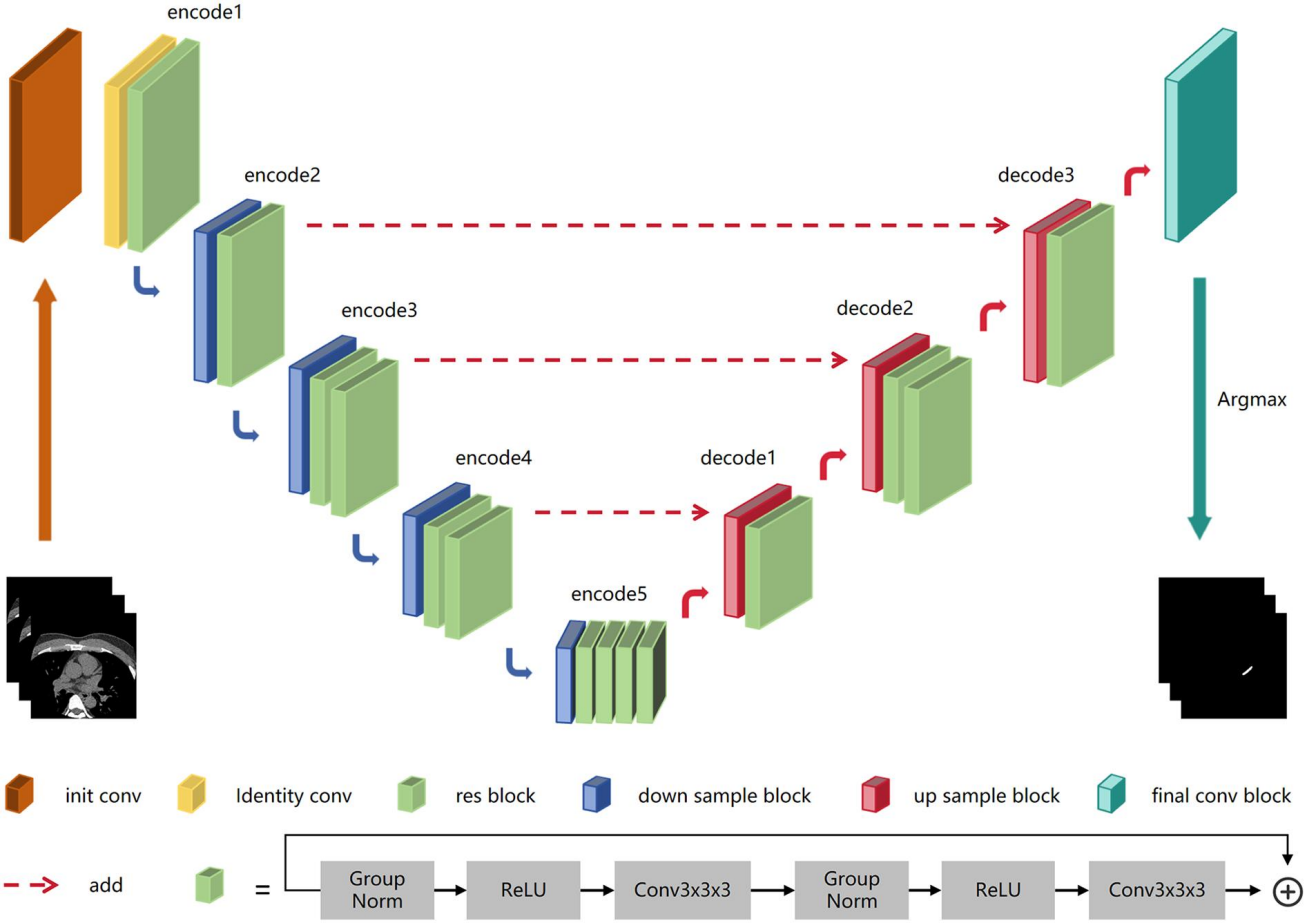
Architecture of the SegResNet model for automatic coronary artery segmentation. The network follows an encoder-decoder structure with residual connections, where the encoder consists of multiple residual blocks (denoted in green), each incorporating group normalization, ReLU activations, and 3 × 3 × 3 convolutions. The encoder progressively downsamples the image, extracting high-level features, while the decoder mirrors the encoder's structure, upsampling using transposed convolutions to reconstruct the segmentation map. Skip connections are used to add the features from the encoder to the corresponding layers in the decoder, enhancing spatial details. The end of the decoder has the same spatial size as the original image, and the final layer uses a softmax function to output classification.

To develop deep learning-based automatic segmentation models, the manually outlined coronary arteries in CACS images from the development set were then used for model training and cross-validation. Three different models (i.e., UnetR, SegResNet, and nnU-Net) were applied separately for LAD and RCA segmentation, with performance evaluated using a combination of metrics, including the Dice coefficient, intersection over union (IoU), and Hausdorff distance. As SegResNet consistently outperformed UnetR and nnU-Net across all metrics, achieving Dice coefficients greater than 0.9, superior IoU values, and the smallest Hausdorff distances for both LAD and RCA (Supplementary Table 1), the SegResNet-based LAD and RCA segmentation models were selected for subsequent automatic delineation of the included arteries across all datasets. The SegResNet is a recently proposed model characterized by a larger encoder for high-level feature extraction and a smaller decoder for refinement (31), with its architecture illustrated in Figure 2. All deep learning operations were implemented using Python with PyTorch and MONAI. Detailed software versions and hardware specifications are provided in Supplementary Table 2.

Following the acquisition of deep learning-based ROIs of the included LAD and RCA arteries in all datasets, the principal steps for extracting the corresponding PCAT ROIs are as follows: (1) The image data was converted to floating-point format and then smoothed to reduce noise; (2) The vessel surface was extracted, the vessel centerline was identified, and the vessel diameters were calculated; (3) For each vessel, the PCAT ROI was then generated by selecting the area with attenuation values between −190 to −30 Hounsfield units, within a radial distance from the outer vessel wall equal to the vessel diameter (18). These procedures were implemented in Python and the VTK library. As a result, the coronary artery masks and PCAT masks for all included coronary arteries in all datasets were finally obtained, and the accuracy was confirmed by one experienced radiologist.

### Radiomics feature extraction and selection

Before feature extraction, gray-level discretization was applied to convert the continuous image to discrete integer values with a bin width of 25. The radiomics features were then extracted by using the PyRadiomics package in Python. For each included LAD or RCA, three kinds of ROI were defined: (1) the coronary artery, (2) PCAT, and (3) a combined region generated by merging the coronary artery and PCAT masks into a single binary ROI. A total of 1,688 radiomics features were extracted from each ROI, which included 324 first-order statistical features, 1,350 texture features, and 14 shape-based features (with the feature counts across different image types aggregated). Wavelet transform images were generated by 8 different combinations of high and low frequency bands in 3 directions (x, y, z). Sigma values of Laplacian of Gaussian filtered images were set to 1, 2, 3, 4, and 5 mm, respectively. Nonlinear strength transformation of image voxel included square, square root, logarithm, and exponential operations.

The development set was subsequently split into a training set (85%) and a validation set (15%), with the stratified sampling method applied to ensure balanced grouping in the training cohort. The feature selection processes were conducted solely on the training set. Specifically, Z-score normalization was applied to eliminate the influence of magnitude differences between features, and the maximum relevance minimum redundancy (mRMR) method was used to initially select 30 features, followed by the application of the least absolute shrinkage and selection operator (LASSO) to further eliminate redundant features. The penalty parameters for LASSO were adjusted using 10-fold cross-validation, and the optimal lambda value was chosen to identify the most appropriate feature subset (Supplementary Figure 2).

### Radiomics model building and validation

A radiomics score (Radscore) was calculated as a linear combination of the selected features from LASSO, weighted by their respective coefficients. Age and sex were not included in the LASSO step but were added afterward in a separate logistic regression together with the Radscore, to enhance prediction performance. Specifically, logistic regression models were built to predict the presence of non-calcified plaques in the coronary arteries, with separate models constructed for LAD and RCA in each of the three regions: (1) coronary artery, (2) PCAT, and (3) the combined region of the coronary artery and PCAT. The training set was used for training each model, with hyperparameter tuning performed through 5-fold cross-validation on the training set. Each model's performance was then assessed for both the training and validation sets. Finally, the internal and external test sets were used to evaluate each model's generalization ability across different patient populations.

### Statistical analysis

All statistical analyses were performed using R software. Descriptive statistics were used to summarize the clinical characteristics. The normality of continuous variables was assessed using the Shapiro–Wilk test. Depending on the normality of the data, two-sample *t*-tests (for normally distributed data) or Mann–Whitney U tests (for non-normally distributed data) were used to compare differences between the lesion and control groups. For categorical variables, chi-square tests were applied. Statistical significance was set at *P* < 0.05.

The performance of the models in each set was estimated using area under the receiver operating characteristic (ROC) curve (AUC), along with accuracy, sensitivity, specificity, positive predictive value (PPV), and negative predictive value (NPV). The AUCs for the three categories—(1) coronary artery, (2) PCAT, and (3) the combined region of the coronary artery and PCAT—were compared pairwise using DeLong tests (32). No correction for multiple comparisons was applied for the AUC comparisons. Decision curve analysis was conducted to assess the net benefit and clinical utility of each model. Statistical significance was considered when *P* < 0.05.

## Results

### Clinical demographics

Table 1 presents the clinical demographics of our study cohort. In total, the training set included 1,212 vessels from 874 patients, the validation set 218 vessels from 203 patients, the internal test set 549 vessels from 388 patients, and the external test set 391 vessels from 280 patients. Across all datasets, patients in the lesion group were significantly older than those in the control group (*P* < 0.001 for the training set, validation set, internal test set, and external test set). Additionally, in all datasets, the proportion of male patients in the lesion group was significantly higher than in the control group (*P* < 0.001 for the training set; *P* = 0.021 for the validation set; *P* < 0.001 for the internal test set; and *P* = 0.002 for the external test set).

**Table 1:** Clinical demographic information from all datasets.

Dataset	Item	Lesion group	Control group	*P*
Training set	*N* of vessels	503	709	–
	LAD	311	366	–
	RCA	192	343	–
	Age, years	58.59 ± 10.93	50.84 ± 11.87	<0.001^a^
	Sex (male/female)	343/160	388/321	<0.001^b^
Validation set	*N* of vessels	89	129	–
	LAD	59	62	–
	RCA	30	67	–
	Age, years	58.25 ± 10.15	51.20 ± 11.16	<0.001^c^
	Sex (male/female)	61/28	67/62	0.021^b^
Internal test set	*N* of vessels	275	274	–
	LAD	131	137	–
	RCA	144	137	–
	Age, years	56.46 ± 8.84	51.12 ± 11.30	<0.001^a^
	Sex (male/female)	185/90	120/154	<0.001^b^
External test set	*N* of vessels	182	209	–
	LAD	126	115	–
	RCA	56	94	–
	Age, years	59.68 ± 11.07	52.61 ± 10.43	<0.001^c^
	Sex (male/female)	141/41	130/79	0.002^b^

Age is presented as mean ± standard deviation, and the other data are presented as frequencies.

*N*, Number; LAD, Left anterior descending artery; RCA, Right coronary artery.

a *P*-value with Mann–Whitney *U*-test.

b *P*-value with chi-square test.

c *P*-value with two-sample *t*-test.

### Deep learning-based segmentation performance

**Figure 3:**
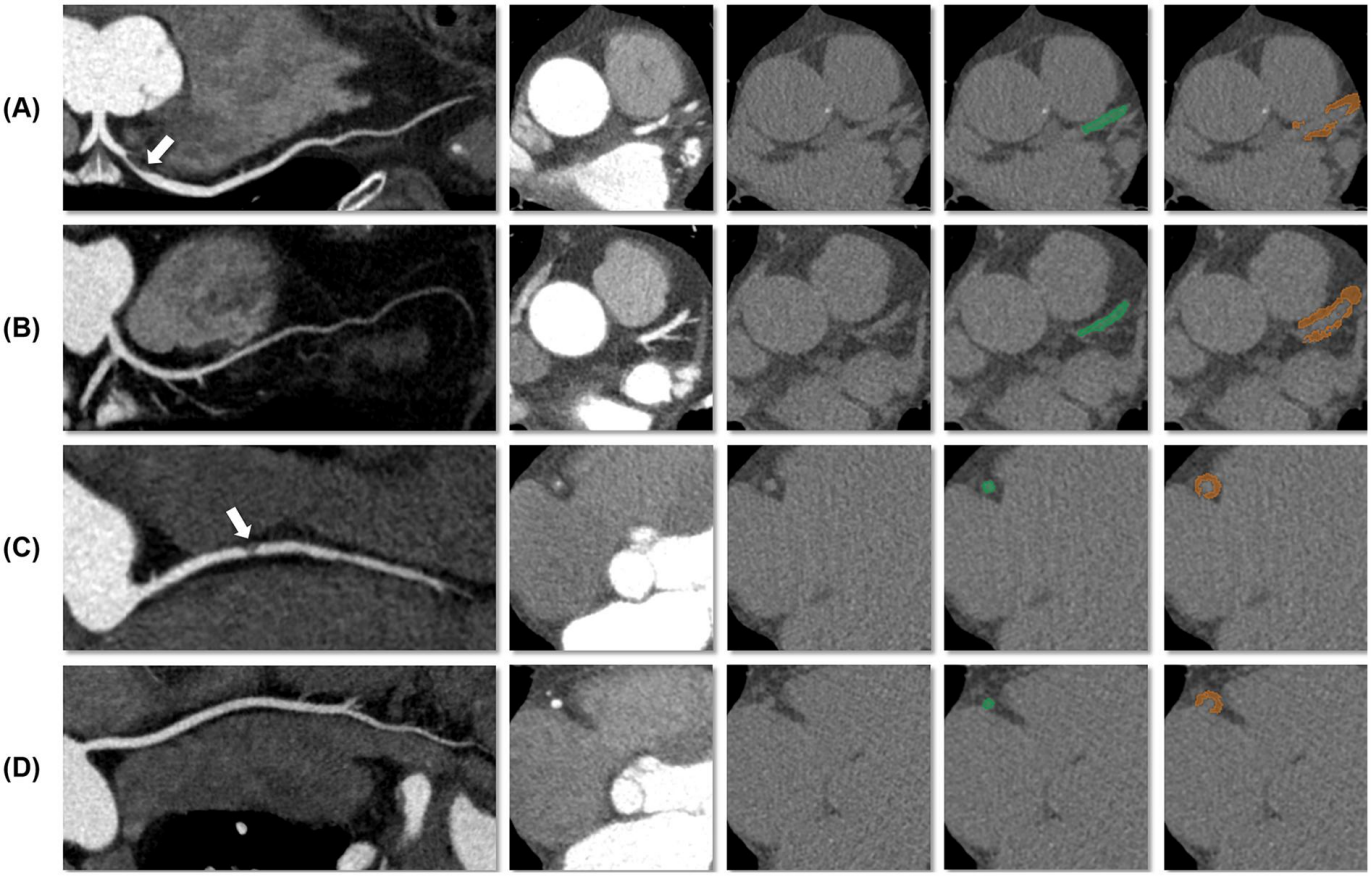
Illustration of deep learning-based segmentation results. Each row represents one case: **(A)** LAD from the lesion group (62-year-old male), **(B)** LAD from the control group (65-year-old male), **(C)** RCA from the lesion group (65-year-old male), and **(D)** RCA from the control group (61-year-old male). From left to right, the columns show: curved planar reformation CCTA image, axial CCTA image, corresponding axial CACS image, coronary artery mask (green) derived from deep learning segmentation overlaid on the CACS image, and PCAT mask (orange) generated based on coronary segmentation overlaid on the CACS image. The white arrow on the curved planar reformation image of the lesion group indicates the location of the non-calcified plaque. LAD, left anterior descending artery; RCA, right coronary artery; CCTA, coronary CT angiography; CACS, coronary artery calcium score; PCAT, pericoronary adipose tissue.

The performance of different deep learning-based models, including UnetR, SegResNet, and nnU-Net, was evaluated for the automatic segmentation of coronary arteries (Supplementary Table 1). Overall, SegResNet demonstrated the highest segmentation accuracy, achieving optimal performance in overlap metrics of Dice (LAD: 0.900 ± 0.061; RCA: 0.908 ± 0.056) and IoU (LAD: 0.840 ± 0.066; RCA: 0.850 ± 0.059). Furthermore, SegResNet excelled in boundary delineation, showing the smallest Hausdorff distances (LAD: 2.998 ± 4.736 mm; RCA: 2.591 ± 1.665 mm). These results suggest the capability of SegResNet to enable automated coronary artery delineation in non-contrast CACS images. Example images of segmentation outcomes for both LAD and RCA from the lesion and control groups are shown in Figure 3.

### Radiomics feature extraction and selection

**Figure 4:**
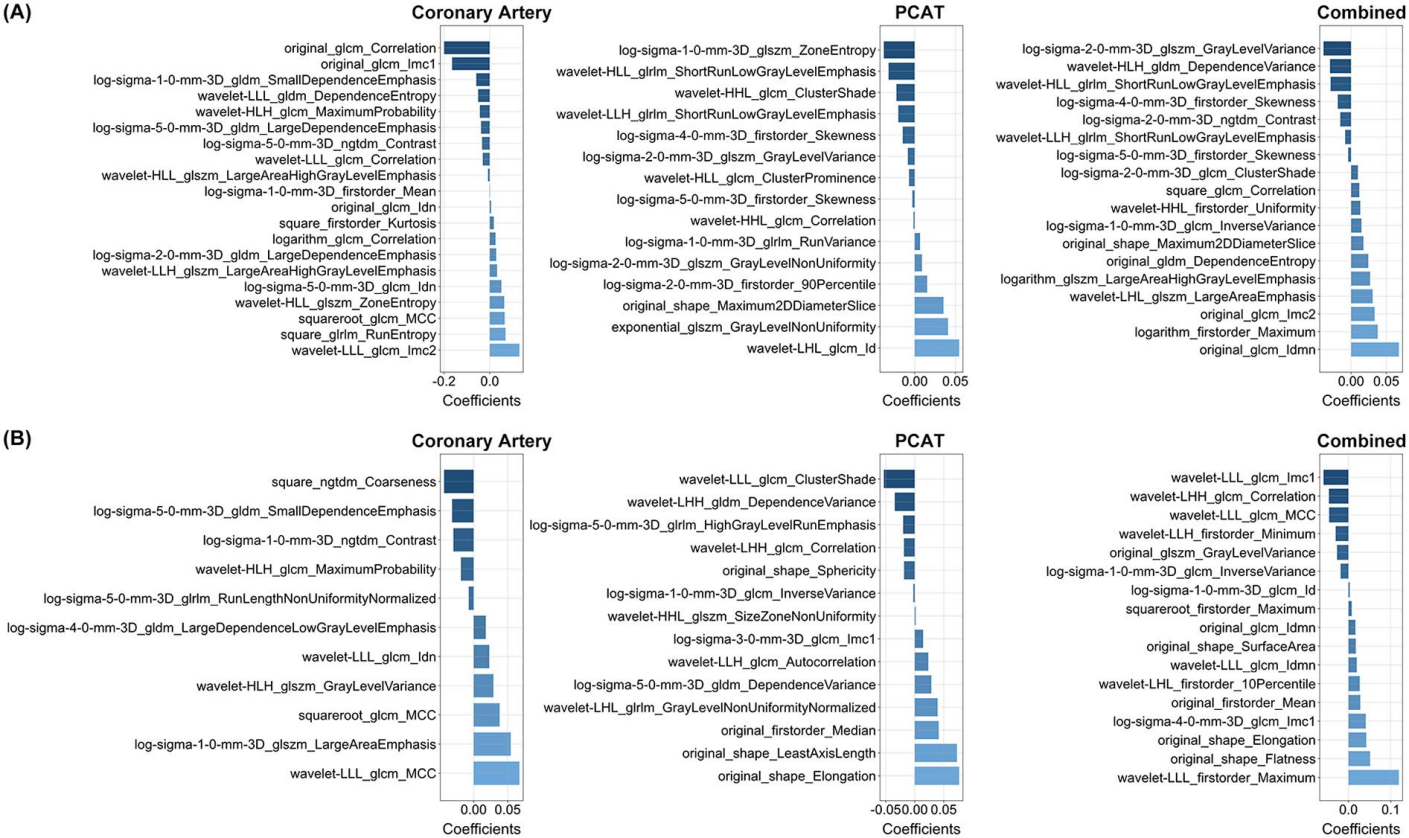
Waterfall plots illustrating the regression coefficients for the selected radiomics features used to predict the presence of non-calcified coronary plaques in the LAD **(A)** and RCA **(B)**. From left to right, each column represents the model derived from the coronary artery alone, PCAT alone, and the combined region of the coronary artery and PCAT, respectively, for both the LAD and RCA. LAD, left anterior descending artery; RCA, right coronary artery; PCAT, pericoronary adipose tissue.

Based on the training set, radiomic features were separately extracted and selected for LAD and RCA across three regions: coronary artery, PCAT, and the combined region of the coronary artery and PCAT. Ultimately, for LAD, we obtained 20 features for coronary artery (18 texture features and 2 first-order features), 15 features for PCAT (11 texture features, 3 first-order features, and 1 shape-based feature), and 18 features for the combined region (13 texture features, 4 first-order features, and 1 shape-based feature). For RCA, we finally obtained 11 features for coronary artery (11 texture features), 14 features for PCAT (10 texture features, 1 first-order feature, and 3 shape-based features), and 17 features for the combined region (9 texture features, 5 first-order features, and 3 shape-based features). Detailed information on the selected radiomic features with their contributions, as measured by the corresponding regression coefficients, is presented in Figure 4.

### Model development and validation

**Figure 5:**
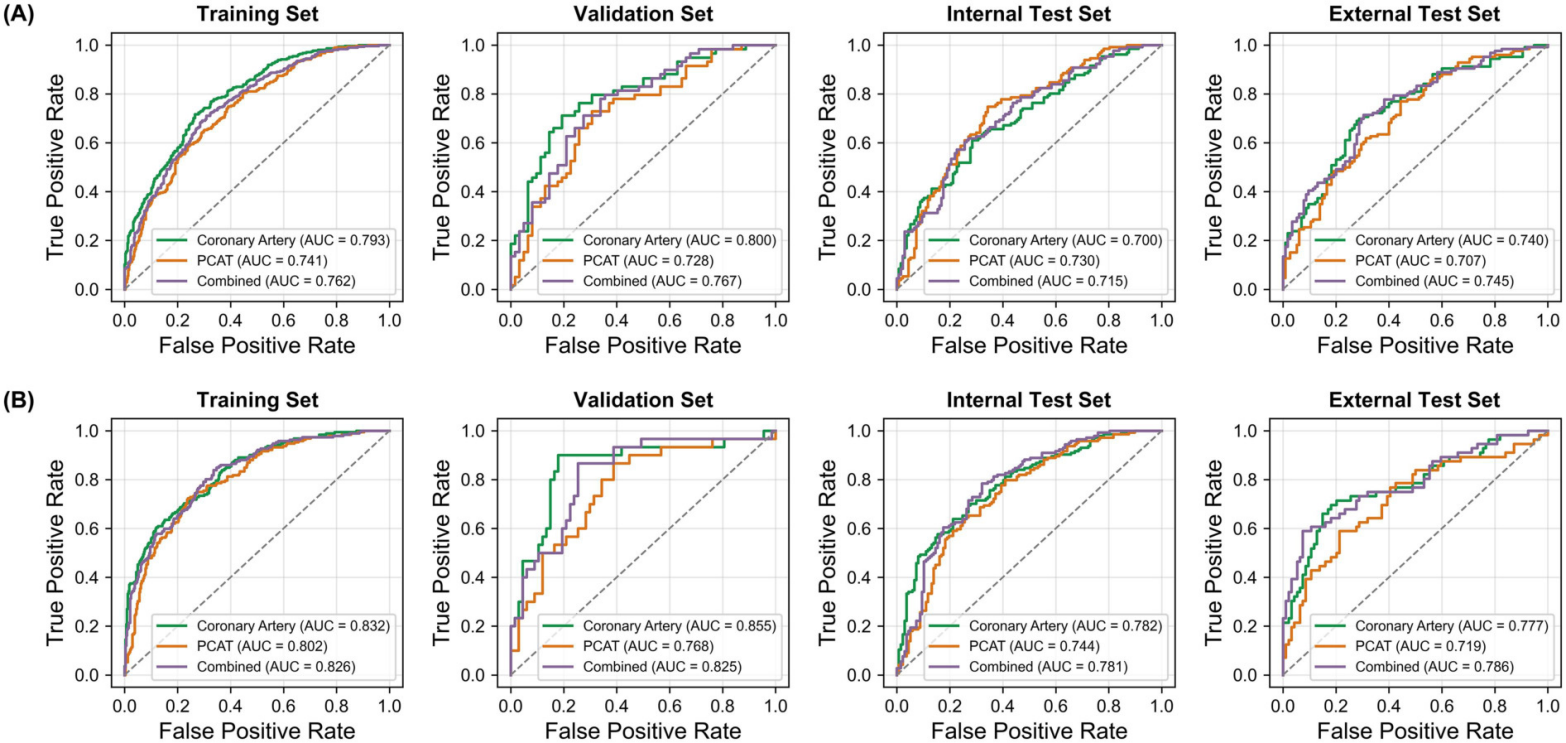
ROC curves for radiomics models predicting non-calcified coronary plaques in the LAD **(A)** and RCA **(B)**. From left to right, each column corresponds to a distinct dataset, i.e., the training set, validation set, internal test set, and external test set. The green, orange, and purple lines represent the models derived from the coronary artery alone, PCAT alone, and the combined region of the coronary artery and PCAT, respectively, for both the LAD and RCA. The AUC values for each model are included in the figure legend of the subplots. ROC, receiver operating characteristic; LAD, left anterior descending artery; RCA, right coronary artery; PCAT, pericoronary adipose tissue; AUC, area under the curve.

**Figure 6:**
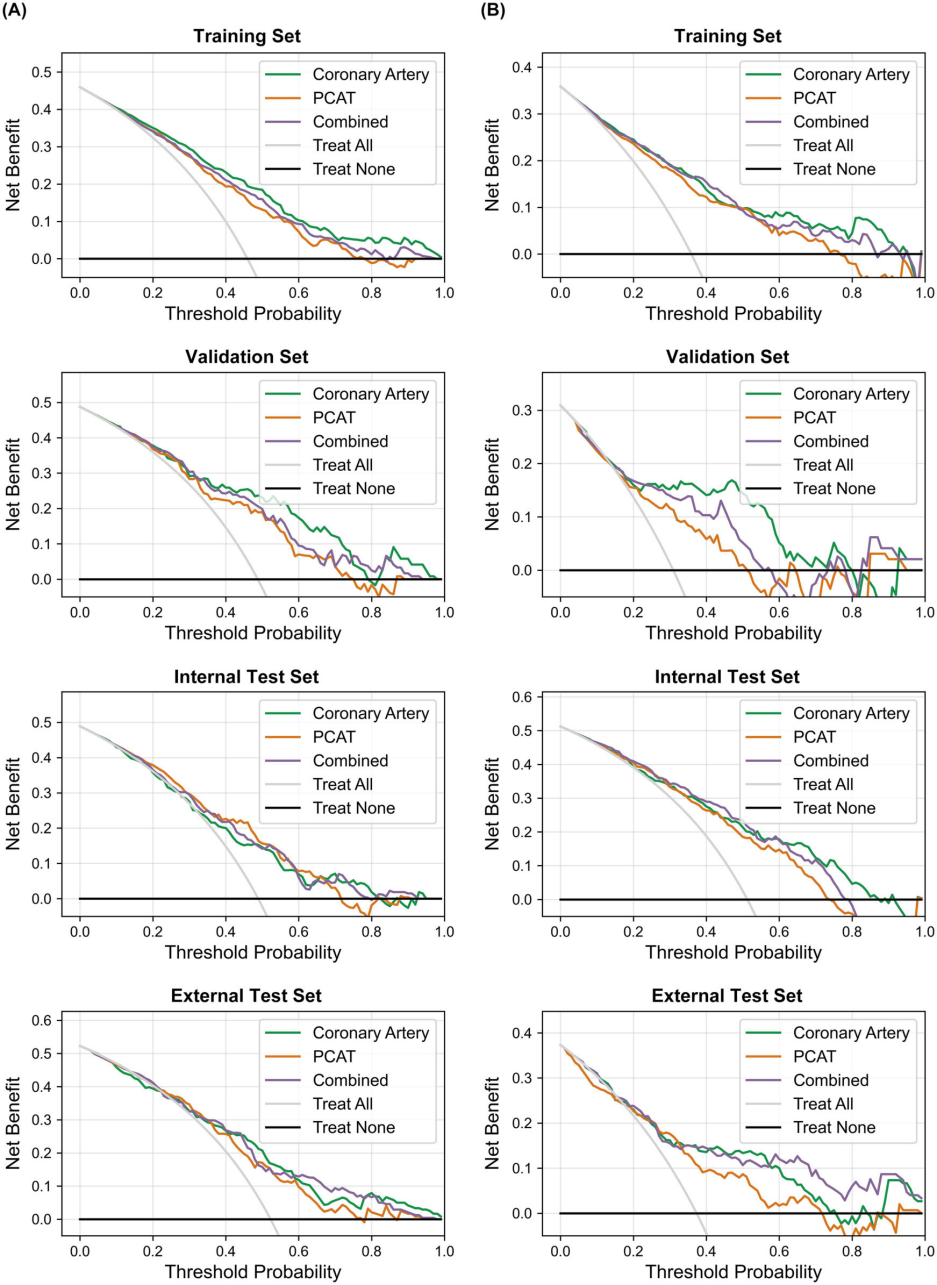
Decision curves of the radiomics models predicting non-calcified coronary plaques in the LAD **(A)** and RCA **(B)**. From top to bottom, each row represents a distinct dataset, i.e., the training set, validation set, internal test set, and external test set. The green, orange, and purple lines represent the models derived from the coronary artery alone, PCAT alone, and the combined region of the coronary artery and PCAT, respectively, for both the LAD and RCA. Overall, all established radiomics models demonstrate good performance for clinical applications across each dataset. LAD, left anterior descending artery; RCA, right coronary artery; PCAT, pericoronary adipose tissue.

The performances of the models for predicting non-calcified plaques were assessed sequentially for the training set, validation set, internal test set, and external test set. In general, the models exhibited moderate to good AUCs across the datasets. For LAD, the coronary artery model achieved AUCs ranging from 0.700 to 0.800, the PCAT model from 0.707 to 0.741, and the combined-region model from 0.715 to 0.767 across the four datasets. For RCA, the coronary artery model ranged from 0.777 to 0.855, the PCAT model from 0.719 to 0.802, and the combined-region model from 0.781 to 0.826. The ROC curves are shown in Figure 5. Detailed AUC values, along with accuracy, sensitivity, specificity, PPV, and NPV, are provided in Table 2. The decision curve analysis suggested good performance of each established model for clinical applications across all datasets (Figure 6).

**Table 2:** Model performance of radiomics models predicting non-calcified coronary plaques across all datasets.

Dataset	Vessel	Region	AUC (95% CI)	Accuracy	Sensitivity	Specificity	PPV	NPV
Training set	LAD	Coronary artery	0.793 (0.760–0.824)	0.725	0.714	0.735	0.696	0.751
		PCAT	0.741 (0.705–0.777)	0.672	0.656	0.686	0.639	0.701
		Combined	0.762 (0.725–0.797)	0.700	0.685	0.713	0.670	0.727
	RCA	Coronary artery	0.832 (0.795–0.867)	0.738	0.714	0.752	0.617	0.824
		PCAT	0.802 (0.762–0.840)	0.738	0.729	0.743	0.614	0.831
		Combined	0.826 (0.788–0.862)	0.735	0.708	0.749	0.613	0.821
Validation set	LAD	Coronary artery	0.800 (0.718–0.875)	0.744	0.746	0.742	0.733	0.754
		PCAT	0.728 (0.633–0.819)	0.702	0.712	0.694	0.689	0.717
		Combined	0.767 (0.683–0.845)	0.711	0.763	0.661	0.682	0.745
	RCA	Coronary artery	0.855 (0.757–0.940)	0.835	0.867	0.821	0.684	0.932
		PCAT	0.768 (0.650–0.874)	0.701	0.700	0.701	0.512	0.839
		Combined	0.825 (0.732–0.913)	0.742	0.667	0.776	0.571	0.839
Internal test set	LAD	Coronary artery	0.700 (0.631–0.761)	0.657	0.626	0.686	0.656	0.657
		PCAT	0.730 (0.672–0.789)	0.668	0.641	0.693	0.667	0.669
		Combined	0.715 (0.654–0.776)	0.653	0.626	0.679	0.651	0.655
	RCA	Coronary artery	0.782 (0.728–0.832)	0.701	0.667	0.737	0.727	0.678
		PCAT	0.744 (0.688–0.801)	0.673	0.694	0.650	0.676	0.669
		Combined	0.781 (0.727–0.835)	0.722	0.715	0.730	0.736	0.709
External test set	LAD	Coronary artery	0.740 (0.675–0.802)	0.685	0.611	0.765	0.740	0.642
		PCAT	0.707 (0.637–0.770)	0.647	0.619	0.678	0.678	0.619
		Combined	0.745 (0.676–0.806)	0.635	0.516	0.765	0.707	0.591
	RCA	Coronary artery	0.777 (0.693–0.853)	0.760	0.696	0.798	0.672	0.815
		PCAT	0.719 (0.627–0.801)	0.713	0.589	0.787	0.623	0.763
		Combined	0.786 (0.710–0.856)	0.733	0.643	0.787	0.643	0.787

The “combined” model refers to the combined-region model, i.e., the model derived from the combined coronary artery and PCAT region.

AUC, Area under the curve; CI, Confidence interval; PPV, Positive predictive value; NPV, Negative predictive value; LAD, Left anterior descending artery; RCA, Right coronary artery; PCAT, Pericoronary adipose tissue.

### Pairwise AUC comparisons for different models

The DeLong test results comparing the AUCs for models derived from distinct regions are presented in Table 3. With reference to the AUC findings, the coronary artery and combined-region models were either significantly superior to or comparable with the PCAT model across all datasets. Regarding the comparisons between the coronary artery and the combined-region models, except for the LAD in the training set (where the coronary artery model outperformed the combined-region model), the AUCs of both models were comparable across all other datasets.

**Table 3:** Pairwise comparison of AUCs for models across three categories (coronary artery, PCAT, and combined region) using DeLong test.

Dataset	Vessel	Region	*P*		
			Coronary artery vs.	PCAT vs.	Combined vs.
Training set	LAD	Coronary artery	–	<0.001	0.006
		PCAT	<0.001	–	0.025
		Combined	0.006	0.025	–
	RCA	Coronary artery	–	0.013	0.465
		PCAT	0.013	–	0.011
		Combined	0.465	0.011	–
Validation set	LAD	Coronary artery	–	0.022	0.186
		PCAT	0.022	–	0.063
		Combined	0.186	0.063	–
	RCA	Coronary artery	–	0.005	0.230
		PCAT	0.005	–	0.035
		Combined	0.230	0.035	–
Internal test set	LAD	Coronary artery	–	0.266	0.517
		PCAT	0.266	–	0.361
		Combined	0.517	0.361	–
	RCA	Coronary artery	–	0.085	0.936
		PCAT	0.085	–	0.004
		Combined	0.936	0.004	–
External test set	LAD	Coronary artery	–	0.161	0.806
		PCAT	0.161	–	0.028
		Combined	0.806	0.028	–
	RCA	Coronary artery	–	0.167	0.704
		PCAT	0.167	–	0.063
		Combined	0.704	0.063	–

The “combined” model refers to the combined-region model, i.e., the model derived from the combined coronary artery and PCAT region.

AUC, Area under the curve; PCAT, Pericoronary adipose tissue; LAD, Left anterior descending artery; RCA, Right coronary artery.

## Discussion

This study presents an automated pipeline for screening non-calcified coronary plaques in the LAD and RCA using the non-contrast CACS sequence, which was validated on datasets from two centers with relatively large sample sizes. Our key findings are: (1) The SegResNet-based deep learning technique enabled the automatic delineation of coronary artery ROIs in CACS images. (2) Models predicting non-calcified plaques (from coronary artery, PCAT, and the combined region) achieved moderate to good performances, with AUCs across datasets ranging from 0.700 to 0.855. (3) The coronary artery and combined-region models performed better than or similarly to the PCAT model, and the AUCs of these two models were comparable except for the LAD in the training set. These results highlight the feasibility of combining deep learning with radiomics based on CACS images for efficient non-calcified plaque detection. Furthermore, given both performance and practicality, we recommend the combined-region models for future clinical use.

The theoretical foundation for detecting non-calcified coronary plaques via CACS sequence builds upon the following pathophysiological and imaging insights. On one hand, the components of non-calcified plaques in the coronary arteries can exhibit distinct CT attenuation values [7–152 Hounsfield unit (HU)] compared to epicardial fat (negative HU values) and blood (approximately 40 HU) (33). Additionally, the formation of non-calcified plaques is often linked to positive vascular remodeling (34). As early as Thilo et al.'s study (13), CACS images revealed visually perceptible features suggesting the presence of non-calcified plaques. On the other hand, PCAT undergoes compositional changes in response to vascular inflammation, which is closely associated with atherosclerosis progression (35). Under inflammatory conditions, pro-inflammatory cytokines released from the vascular wall affect PCAT through paracrine signaling, inhibiting preadipocyte differentiation and lipid accumulation (17–20). Vascular inflammation also induces irreversible changes in PCAT, such as fibrosis and microvascular remodeling (20, 36). These PCAT alterations lead to measurable changes in tissue density that can be captured on CT images and systematically analyzed using radiomics.

In our study, following feature selection, the retained radiomic features for model construction included several first-order, texture, and shape-based metrics. First-order statistics describe the intensity distribution of voxels within an ROI without accounting for spatial dependencies (37), while texture features capture spatial relationships revealing heterogeneity information (38), and shape-based features represent the geometric properties of the ROI (39). Notably, texture metrics predominated in each model, regardless of whether based on coronary artery, PCAT, or their combined region. For arteries, a similar finding was reported by Wang et al. (39), showing that texture features helped distinguish non-calcified carotid plaques from the absence of plaques. This result is plausible, as changes associated with non-calcified plaques can indeed lead to regional heterogeneity across the vessel ROI. For PCAT, the identification of texture features also aligns with prior investigations (25, 26, 40, 41), which may indicate fibrosis and vascularity, reflecting the persistent alterations in adipose tissue due to chronic coronary inflammation (29). Taken together, our findings suggest that radiomics could offer a thorough depiction of non-calcified plaque features.

The radiomic models based on CACS for predicting non-calcified coronary plaques achieved moderate to good performance, with AUCs ranging from 0.700 to 0.855. These values are within the range of those reported in previous studies. For example, Kruk et al. reported an AUC of 0.67 (14), Jiang et al. reported 0.896 (25), and Yu et al. reported 0.963–0.998 (26). However, direct cross-study comparison is difficult because of potential differences in population characteristics (e.g., CAD prevalence, proportion of non-calcified plaques), imaging acquisition protocols, ROI definitions, and modeling pipelines. The observed variations in AUCs across studies should therefore be interpreted with caution and may partly reflect these methodological and population differences. Given that future studies may incorporate more comprehensive clinical data and advanced modeling techniques, we believe the performance reported here represents a baseline, with potential for better outcomes in future investigations. Additionally, our findings revealed that the radiomic models performed more effectively in the RCA compared to the LAD across all model types (i.e., coronary artery, PCAT, and the combined-region models). We speculate that this phenomenon may be related to differences in plaque distribution (42) and the amount of perivascular fat content (25) between the LAD and RCA.

Extending prior research in the field, this study systematically evaluated three modeling strategies—based on the coronary artery, PCAT, and their combined region—for performance comparisons. The results indicated that both the coronary artery and combined-region models outperformed or matched the performance of the PCAT model, while the capabilities of the former two models were equivalent in most conditions. An interpretation is provided below. First, the favorable performance of the coronary artery model supports the diagnostic value of coronary artery features from non-contrast CT in identifying non-calcified plaques, aligning with findings reported in prior literature (14, 43). Second, the combined-region model generally exhibited non-inferior diagnostic capability. This may be explained by the inclusion of both direct plaque-specific information from the coronary artery and indirect inflammation-related information from PCAT. Considering the inherent difficulty of delineating coronary arteries on non-contrast CT in real-world settings, our findings support the use of a combined coronary artery and PCAT segmentation approach in future investigations. This conceptual innovation offers practical guidance by improving analytical efficiency and enhancing feasibility, which could potentially accelerate the dissemination of related research and its eventual clinical translation.

Screening for non-calcified plaques in the coronary arteries is vital for cardiovascular risk assessment, as these plaques are prone to rupture and thrombosis (44). The SCOT-HEART trial found that low-attenuation plaque burden serves as the strongest predictor of cardiovascular outcomes, which is independent of calcium score (7). Our pipeline for primarily screening of non-calcified coronary plaques using non-contrast CT may potentially serve as a supportive or triage tool to guide suspected patients toward CCTA following an initial diagnosis, thereby optimizing the workflow for more targeted evaluation. This approach may enhance the positive detection rate and pertinence of CCTA, while reducing unnecessary contrast agent use and the associated risk of allergic reactions, conserving medical resources, and potentially alleviating overall healthcare burdens. Furthermore, we innovatively leverage manually delineated data to build a SegResNet-based deep learning model, enabling automatic delineation of coronary arteries in non-contrast CT images for unseen data. This automation streamlines the workflow, overcoming the challenges of manual delineation and enhancing feasibility for broader clinical adoption.

To our knowledge, although the current work may be the largest study to date using non-contrast CACS for screening non-calcified coronary plaques, several limitations should be acknowledged. First, to maintain a sufficiently large sample size and ensure model robustness and generalizability, our study was unable to incorporate detailed clinical factors—particularly cardiovascular risk factors (e.g., BMI, history of hypertension, diabetes, and smoking)—due to incomplete data. Instead, we included only age and gender, the two clinical indicators that can be definitively obtained during CT examination. The absence of these risk factors in the current study may have constrained model performance by limiting the characterization of individual clinical risk profiles. Second, more advanced modeling techniques were not applied, which could potentially contribute to better outcomes. Third, the current analysis is limited to the LAD and RCA; the LCX was excluded due to its variable anatomy, small calibre, tortuous path, and limited surrounding adipose tissue (11, 29). Nevertheless, the LCX is clinically important: left dominant circulation and isolated LCX disease are not rare, and their exclusion limits the generalizability of our models. Future work should focus on optimizing segmentation and feature extraction strategies to support robust LCX assessment. Fourth, considering that thicker reconstructions may increase partial-volume effects, the CACS scans in this study were reconstructed with a slice thickness of 0.625 mm (consistent with CCTA) to ensure the reliability of this investigation. For clinical translation, validation of the reproducibility in routine clinical reconstruction settings is warranted. Fifth, identical equipment and protocols were used across centers to ensure uniformity, which restricts understanding of model performance on other scanners or in different clinical settings. Future multi-scanner and real-world studies are needed to evaluate generalizability. Sixth, the present study focused on vessel-level analysis, as patient-level prediction may face methodological challenges, including concerns related to vessel-level aggregation, and potential confounding by contralateral calcified plaques in direct patient-level modeling. Future methodological development is needed to enable robust patient-level prediction. Seventh, it is important to emphasize that our model is fundamentally exploratory and is intended as a screening or triage tool, rather than as a definitive diagnostic test. Clinical decisions should continue to be guided by comprehensive evaluation, including CCTA or invasive imaging when appropriate. Last, the study used ECG-gated CACS instead of routine chest CT scans, as the latter faces challenges from cardiac motion artifacts. Should this issue be addressed, and with chest CT becoming standard in health check-ups (45), it could lead to a significant breakthrough in achieving dual screening for both lung cancer and CAD using a single chest CT scan.

In conclusion, by combining deep learning with radiomics, this study introduces an automated pipeline for screening non-calcified coronary plaques in the LAD and RCA via the non-contrast CACS sequence, validated with relatively large samples. The models achieved moderate to good predictive performances, supporting the feasibility of this approach for efficient detection. Additionally, the combined-region models show promise for future application. Our findings provide a preliminary basis for the potential use of chest CT scans in large-scale CAD screening in the future.

## Data Availability

The raw data supporting the conclusions of this article will be made available by the authors, without undue reservation.
