# Supplementary material for: Automated deep learning–radiomics pipeline for non-calcified coronary plaque detection using non-contrast calcium score CT
Source: Front Cardiovasc Med. 2026 Jun 1;13:1794024. doi: 10.3389/fcvm.2026.1794024 (PMC13265288; doi:10.3389/fcvm.2026.1794024)
Supplement: Supplementary file 1 [file Datasheet1.docx]

***Supplementary Material***

**
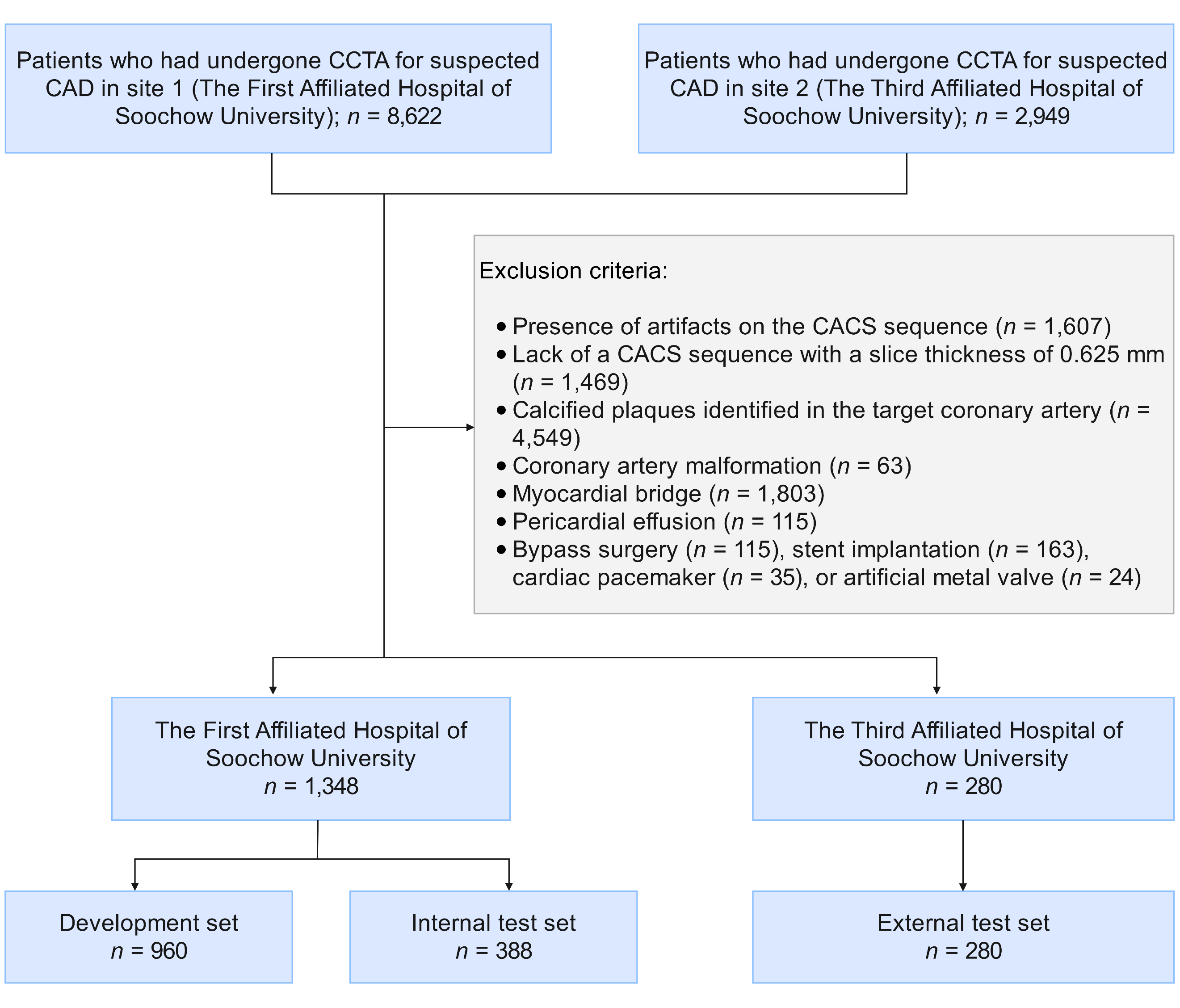
**

**Supplementary Figure 1.** Flowchart of study patient enrolment.

CCTA, coronary CT angiography; CAD, coronary artery disease; *n*, number of subjects; CACS, coronary artery calcium score.

**
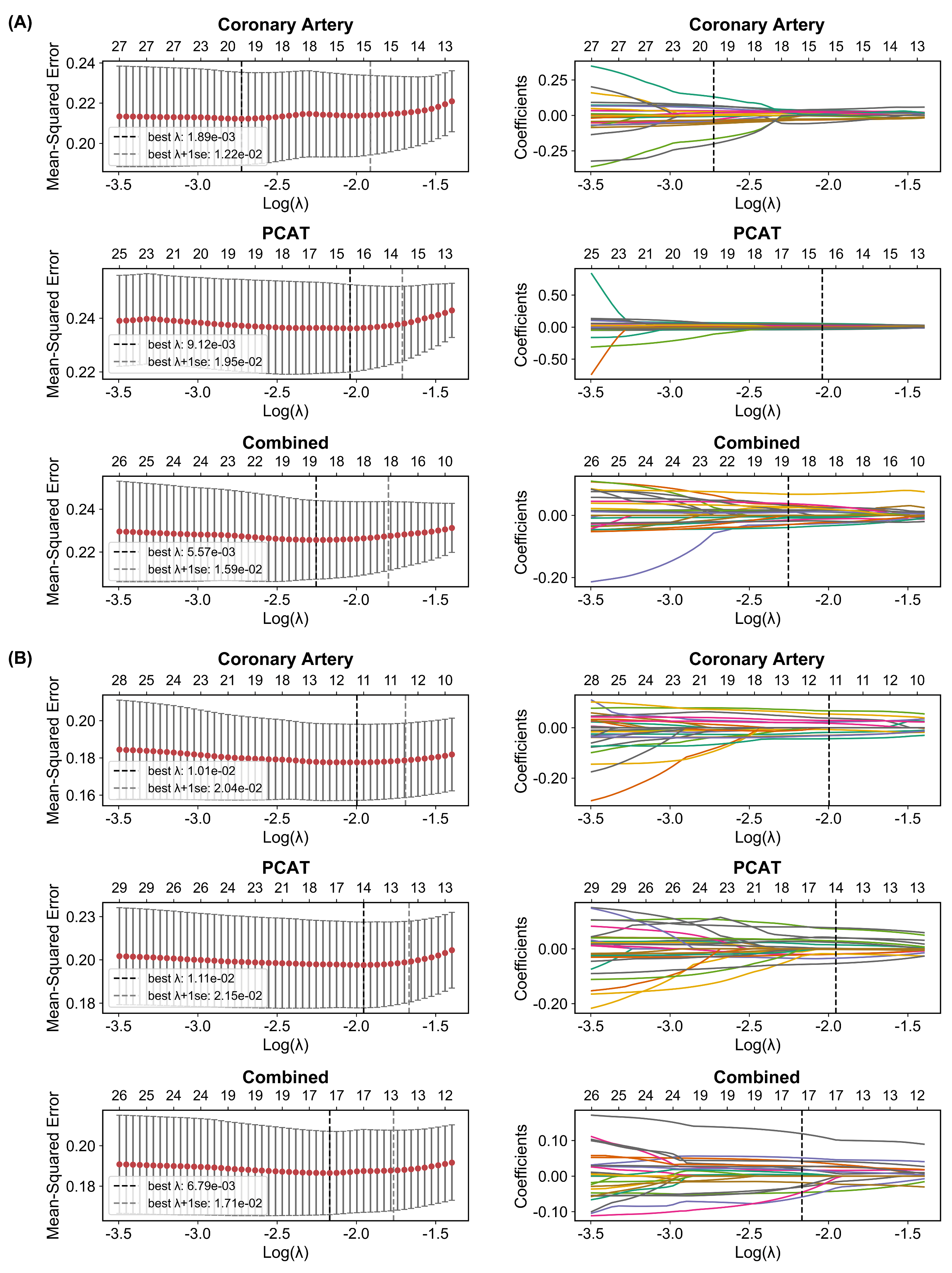
**

**Supplementary Figure 2.** LASSO-based feature selection process. For both the LAD (A) and RCA (B), from top to bottom, each row represents the model derived from the coronary artery alone, PCAT alone, and the combined region of the coronary artery and PCAT, respectively. The left column depicts the selection of penalty parameter lambda through 10-fold cross-validation, where the red dots represent the MSE corresponding to each lambda value, and the grey vertical bars indicate the confidence interval within one standard error of the MSE at that lambda. The black dashed line denotes the optimal lambda with the minimum MSE, while the grey dashed line indicates the lambda at one standard error beyond the minimum MSE. The right column shows the convergence plot of feature coefficients in LASSO regression, with the curve illustrating how the coefficients change as lambda varies. In each subplot, the lower x-axis represents the logarithmic values of lambda, and the upper x-axis shows the number of features with non-zero coefficients corresponding to different lambda values in the model.

LASSO, least absolute shrinkage and selection operator; LAD, left anterior descending artery; RCA, right coronary artery; PCAT, pericoronary adipose tissue; MSE, mean squared error.

**Supplementary Table 1.** Performance metrics for automatic segmentation of LAD and RCA with different deep learning-based models on the development set

| **Model** | **Dice** | **IoU** | **HD (mm)** |
| --- | --- | --- | --- |
| **LAD:** |  |  |  |
| UnetR | 0.863 ± 0.061 | 0.791 ± 0.063 | 27.183 ± 25.528 |
| SegResNet | 0.900 ± 0.061 | 0.840 ± 0.066 | 2.998 ± 4.736 |
| nnUnet | 0.842 ± 0.062 | 0.766 ± 0.061 | 27.879 ± 25.307 |
| **RCA:** |  |  |  |
| UnetR | 0.849 ± 0.049 | 0.772 ± 0.056 | 52.800 ± 33.249 |
| SegResNet | 0.908 ± 0.056 | 0.850 ± 0.059 | 2.591 ± 1.665 |
| nnUnet | 0.867 ± 0.059 | 0.795 ± 0.062 | 15.709 ± 24.011 |

LAD, left anterior descending artery; RCA, right coronary artery; IoU, intersection over union; HD, Hausdorff distance

**Supplementary Table 2.** Software versions and computing environment

| **Category** | **Item** | **Version / Specification** |
| --- | --- | --- |
| Operating system | Ubuntu | 20.04 LTS |
| Hardware | CPU | Intel Xeon Platinum 8375C |
|  | GPU | NVIDIA GeForce RTX 4090 |
|  | RAM | 128 GB |
| Programming language | Python | 3.8.10 |
| Deep learning framework | PyTorch | 1.13.1+cu117 |
|  | MONAI | 1.2.0 |
| Radiomics | PyRadiomics | 3.0.1 |
| Image processing | VTK | 9.0.1 |
| Machine learning | scikit-learn | 1.0.2 |
|  | numpy | 1.21.6 |
|  | pandas | 1.3.5 |
| Statistical analysis | R | 3.5.1 |
